# Variation in general supportive and preventive intensive care management of traumatic brain injury: a survey in 66 neurotrauma centers participating in the Collaborative European NeuroTrauma Effectiveness Research in Traumatic Brain Injury (CENTER-TBI) study

**DOI:** 10.1186/s13054-018-2000-6

**Published:** 2018-04-13

**Authors:** Jilske A. Huijben, Victor Volovici, Maryse C. Cnossen, Iain K. Haitsma, Nino Stocchetti, Andrew I. R. Maas, David K. Menon, Ari Ercole, Giuseppe Citerio, David Nelson, Suzanne Polinder, Ewout W. Steyerberg, Hester F. Lingsma, Mathieu van der Jagt, CENTER-TBI investigators and participants, Hadie Adams, Masala Alessandro, Judith Allanson, Krisztina Amrein, Norberto Andaluz, Nada Andelic, Nanni Andrea, Lasse Andreassen, Audny Anke, Anna Antoni, Hilko Ardon, Gérard Audibert, Kaspars Auslands, Philippe Azouvi, Camelia Baciu, Andrew Bacon, Rafael Badenes, Trevor Baglin, Ronald Bartels, Pál Barzó, Ursula Bauerfeind, Ronny Beer, Javier Francisco Belda, Bo-Michael Bellander, Antonio Belli, Rémy Bellier, Habib Benali, Thierry Benard, Maurizio Berardino, Luigi Beretta, Christopher Beynon, Federico Bilotta, Harald Binder, Erta Biqiri, Morten Blaabjerg, Stine Lund Borgen, Pierre Bouzat, Peter Bragge, Alexandra Brazinova, Felix Brehar, Camilla Brorsson, Andras Buki, Monika Bullinger, Veronika Bučková, Emiliana Calappi, Peter Cameron, Guillermo Lozano Carbayo, Elsa Carise, K. Carpenter, Ana M. Castaño-León, Francesco Causin, Giorgio Chevallard, Arturo Chieregato, Giuseppe Citerio, Maryse Cnossen, Mark Coburn Coburn, Jonathan Coles, Jamie D. Cooper, Marta Correia, Amra Covic, Nicola Curry, Endre Czeiter, Marek Czosnyka, Claire Dahyot-Fizelier, François Damas, Pierre Damas, Helen Dawes, Véronique De Keyser, Francesco Corte Della, Bart Depreitere, Shenghao Ding, Diederik Dippel, Kemal Dizdarevic, Loup Guy Dulière, Adelaida Dzeko, George Eapen, Heiko Engemann, Ari Ercole, Patrick Esser, Erzsébet Ezer, Martin Fabricius, Valery L. Feigin, Junfeng Feng, Kelly Foks, Francesca Fossi, Gilles Francony, Janek Frantzén, Ulderico Freo, Shirin Frisvold, Alex Furmanov, Gagliardo Pablo, Damien Galanaud, Guoyi Gao, Karin Geleijns, Alexandre Ghuysen, Benoit Giraud, Ben Glocker, Pedro A. Gomez, Francesca Grossi, Russell L. Gruen, Deepak Gupta, Juanita A. Haagsma, Ermin Hadzic, Iain Haitsma, Jed A. Hartings, Raimund Helbok, Eirik Helseth, Daniel Hertle, Sean Hill, Astrid Hoedemaekers, Stefan Hoefer, Peter J. Hutchinson, Asta Kristine Håberg, Bram Jacobs, Ivan Janciak, Koen Janssens, Ji Yao Jiang, Kelly Jones, Jean Pierre Kalala, Konstantinos Kamnitsas, Mladen Karan, Jana Karau, Ari Katila, Maija Kaukonen, David Keeling, Thomas Kerforne, Naomi Ketharanathan, Johannes Kettunen, Riku Kivisaari, Angelos G. Kolias, Bálint Kolumbán, Erwin Kompanje, Daniel Kondziella, Lars Owe Koskinen, Noémi Kovács, Ferenc Kálovits, Alfonso Lagares, Linda Lanyon, Steven Laureys, Martin Lauritzen, Fiona Lecky, Christian Ledig, Rolf Lefering, Valerie Legrand, Jin Lei, Leon Levi, Roger Lightfoot, Hester Lingsma, Dirk Loeckx, Angels Lozano, Roger Luddington, Chantal Luijten-Arts, Andrew I. R. Maas, Stephen MacDonald, Charles MacFayden, Marc Maegele, Marek Majdan, Sebastian Major, Alex Manara, Pauline Manhes, Geoffrey Manley, Didier Martin, Costanza Martino, Armando Maruenda, Hugues Maréchal, Dagmara Mastelova, Julia Mattern, Catherine McMahon, Béla Melegh, David Menon, Tomas Menovsky, Cristina Morganti-Kossmann, Davide Mulazzi, Manuel Mutschler, Holger Mühlan, Ancuta Negru, David Nelson, Eddy Neugebauer, Virginia Newcombe, Quentin Noirhomme, József Nyirádi, Mauro Oddo, Annemarie Oldenbeuving, Matej Oresic, Fabrizio Ortolano, Aarno Palotie, Paul M. Parizel, Adriana Patruno, Jean-François Payen, Natascha Perera, Vincent Perlbarg, Paolo Persona, Wilco Peul, Nicolas Pichon, Henning Piilgaard, Anna Piippo, Sébastien Floury Pili, Matti Pirinen, Horia Ples, Suzanne Polinder, Inigo Pomposo, Marek Psota, Pim Pullens, Louis Puybasset, Arminas Ragauskas, Rahul Raj, Malinka Rambadagalla, Veronika Rehorčíková, Jonathan Rhodes, Sylvia Richardson, Samuli Ripatti, Saulius Rocka, Nicolas Rodier, Cecilie Roe, Olav Roise, Gerwin Roks, Pauline Romegoux, Jonathan Rosand, Jeffrey Rosenfeld, Christina Rosenlund, Guy Rosenthal, Rolf Rossaint, Sandra Rossi, Tim Rostalski, Daniel Rueckert, Felix Ruiz de Arcaute, Martin Rusnák, Marco Sacchi, Barbara Sahakian, Juan Sahuquillo, Oliver Sakowitz, Francesca Sala, Paola Sanchez-Pena, Renan Sanchez-Porras, Janos Sandor, Edgar Santos, Nadine Sasse, Luminita Sasu, Davide Savo, Inger Schipper, Barbara Schlößer, Silke Schmidt, Annette Schneider, Herbert Schoechl, Guus Schoonman, Frederik Rico Schou, Elisabeth Schwendenwein, Michael Schöll, Özcan Sir, Toril Skandsen, Lidwien Smakman, Dirk Smeets, Peter Smielewski, Abayomi Sorinola, Emmanuel Stamatakis, Simon Stanworth, Katrin Stegemann, Nicole Steinbüchel, Robert Stevens, William Stewart, Ewout W. Steyerberg, Nino Stocchetti, Nina Sundström, Anneliese Synnot, József Szabó, Jeannette Söderberg, Silvio Fabio Taccone, Viktória Tamás, Päivi Tanskanen, Alexandru Tascu, Mark Steven Taylor, Braden Ao Te, Olli Tenovuo, Guido Teodorani, Alice Theadom, Matt Thomas, Dick Tibboel, Christos Tolias, Luaba Tshibanda Jean-Flory, Maria Cristina Tudora, Peter Vajkoczy, Egils Valeinis, Wim Van Hecke, Dominique Van Praag, Dirk Van Roost, Eline Van Vlierberghe, Thijs Vande Vyvere, Audrey Vanhaudenhuyse, Alessia Vargiolu, Emmanuel Vega, Jan Verheyden, Paul M. Vespa, Anne Vik, Rimantas Vilcinis, Giacinta Vizzino, Carmen Vleggeert-Lankamp, Victor Volovici, Peter Vulekovic, Zoltán Vámos, Derick Wade, Kevin K. W. Wang, Lei Wang, Eno Wildschut, Guy Williams, Lisette Willumsen, Adam Wilson, Lindsay Wilson, Maren K. L. Winkler, Peter Ylén, Alexander Younsi, Menashe Zaaroor, Zhiqun Zhang, Zelong Zheng, Fabrizio Zumbo, Stefanie de Lange, Godard C. W. de Ruiter, Hugo den Boogert, Jeroen van Dijck, Thomas A. van Essen, Caroline van Heugten, Mathieu van der Jagt, Joukje van der Naalt

**Affiliations:** 1 Center for Medical Decision Making, Department of Public Health, Erasmus Medical Center Rotterdam, Rotterdam, the Netherlands; 2 Department of Neurosurgery, Office H-703, Erasmus MC Stroke Center and Brain Tumor Center, Erasmus Medical Center Rotterdam, Rotterdam, The Netherlands; 3 Department of Pathophysiology and Transplants, University of Milan, Milan, Italy; 4 Fondazione IRCCS Ca’ Granda, Ospedale Maggiore Policlinico, Department of Anesthesia and Critical Care, Neuroscience Intensive Care Unit, Milan, Italy; 5 Department of Neurosurgery, Antwerp University Hospital and University of Antwerp, Edegem, Belgium; 6 Division of Anaesthesia, University of Cambridge, Addenbrooke’s Hospital, Cambridge, UK; 7 School of Medicine and Surgery, University of Milan-Bicocca, Milan, Italy; 8 Neurointensive Care, San Gerardo Hospital, ASST-Monza, Monza, Italy; 9 Section for Perioperative Medicine and Intensive Care, Department of Physiology and Pharmacology, Karolinska Institutet, Stockholm, Sweden; 10 Department of Medical Statistics and Bioinformatics, Leiden University Medical Center, Leiden, the Netherlands; 11 Department of Intensive Care and Erasmus MC Stroke Center, Erasmus Medical Center, Rotterdam, the Netherlands

**Keywords:** Intensive care unit, Traumatic brain injury, Glucose, Nutrition, Fever, Ventilation, Blood pressure, Seizure, Survey, Europe

## Abstract

**Background:**

General supportive and preventive measures in the intensive care management of traumatic brain injury (TBI) aim to prevent or limit secondary brain injury and optimize recovery. The aim of this survey was to assess and quantify variation in perceptions on intensive care unit (ICU) management of patients with TBI in European neurotrauma centers.

**Methods:**

We performed a survey as part of the Collaborative European NeuroTrauma Effectiveness Research in Traumatic Brain Injury (CENTER-TBI) study. We analyzed 23 questions focused on: 1) circulatory and respiratory management; 2) fever control; 3) use of corticosteroids; 4) nutrition and glucose management; and 5) seizure prophylaxis and treatment.

**Results:**

The survey was completed predominantly by intensivists (*n* = 33, 50%) and neurosurgeons (*n* = 23, 35%) from 66 centers (97% response rate).

The most common cerebral perfusion pressure (CPP) target was > 60 mmHg (*n* = 39, 60%) and/or an individualized target (*n* = 25, 38%). To support CPP, crystalloid fluid loading (*n* = 60, 91%) was generally preferred over albumin (*n* = 15, 23%), and vasopressors (*n* = 63, 96%) over inotropes (*n* = 29, 44%). The most commonly reported target of partial pressure of carbon dioxide in arterial blood (PaCO_2_) was 36–40 mmHg (4.8–5.3 kPa) in case of controlled intracranial pressure (ICP) < 20 mmHg (*n* = 45, 69%) and PaCO_2_ target of 30–35 mmHg (4–4.7 kPa) in case of raised ICP (*n* = 40, 62%). Almost all respondents indicated to generally treat fever (*n* = 65, 98%) with paracetamol (*n* = 61, 92%) and/or external cooling (*n* = 49, 74%). Conventional glucose management (*n* = 43, 66%) was preferred over tight glycemic control (*n* = 18, 28%). More than half of the respondents indicated to aim for full caloric replacement within 7 days (*n* = 43, 66%) using enteral nutrition (*n* = 60, 92%). Indications for and duration of seizure prophylaxis varied, and levetiracetam was mostly reported as the agent of choice for both seizure prophylaxis (*n* = 32, 49%) and treatment (*n* = 40, 61%).

**Conclusions:**

Practice preferences vary substantially regarding general supportive and preventive measures in TBI patients at ICUs of European neurotrauma centers. These results provide an opportunity for future comparative effectiveness research, since a more evidence-based uniformity in good practices in general ICU management could have a major impact on TBI outcome.

**Electronic supplementary material:**

The online version of this article (10.1186/s13054-018-2000-6) contains supplementary material, which is available to authorized users.

## Background

Traumatic brain injury (TBI) is one of the major causes of trauma-related death and hospital admissions in Europe [1]. TBI is recognized as a complex heterogeneous syndrome [2]. The higher vulnerability of this population is reflected by higher mortality rates in patients with TBI compared with non-head injured trauma patients [3]. Therefore, patients with (severe) TBI require specialized neurointensive care (treatment) at an intensive care unit (ICU) [4].

Case fatality rates in severe TBI are high, ranging from 30% to 40% in unselected observational series [5]. Furthermore, substantial between-country [1] and between-center differences [3, 4, 6] in overall TBI mortality rates exist which might be partly explained by differences in treatment [7–9].

The key objectives of ICU TBI management are to maintain general physiology and prevent secondary brain injury. A number of brain-specific therapies, such as intracranial pressure (ICP)-guided treatment or, less often, brain-metabolic or cerebral vascular autoregulation-based goals are employed both clinically or as the subject of clinical research [10]. However, general support of the cardiovascular system, respiratory function, and nutritional or metabolic needs must not be overlooked and could also have a significant impact on outcome [11, 12]. Cerebral metabolic control by seizure or fever management may further contribute to better outcomes [2, 13–15]. At present, optimal strategies for general management are only partly established [16, 17]. This lack of robust evidence may ultimately result in institutional or individual variations in practice that may contribute to variances in outcome.

The aim of this survey study was to assess variation in ICU management perceptions of general supportive and preventive care policies (including, for instance, circulatory and respiratory management) in patients with TBI in European neurotrauma centers.

## Methods

### Participating centers

This study is part of the Collaborative European NeuroTrauma Effectiveness Research in Traumatic Brain Injury (CENTER-TBI) study that collects data on patient characteristics, patient management, and outcomes in 68 centers from 20 countries across Europe and Israel [18]. All these centers were asked to complete a ‘Provider Profiling Questionnaire’ [19]. The questionnaire items used for this study (treatment at the intensive care) are attached as Additional file 1.

### Provider profiling questionnaire

The provider profiling questionnaire was developed in several stages. First, literature was explored for evidence, including guidelines and available surveys. Second, a pilot study was conducted in 16 participating centers to receive feedback, to determine ambiguity, and to detect unexpected and missing values. Throughout all stages, experts of various disciplines (neurosurgeons, intensivists, neurologists, emergency department physicians, rehabilitation physicians, medical ethicists, health care economists, and epidemiologists) were asked for their advice on the development of the questionnaire. Details on the development, administration, and content of the complete provider profiling questionnaires have been published previously [19].

### General supportive and preventive management

For the purpose of the current study, we focused on 23 questions specifically aimed at general ICU policies (Additional file 1). Specifically, we focused on circulatory and respiratory management, fever control, use of corticosteroids, glucose and nutrition management, and seizure prophylaxis and treatment. Most questions were multiple-choice, except for two questions: the aim for caloric intake in TBI patients and the use of corticosteroids for other conditions. Overall, the general policy of a center rather than the individual treatment preference of the respondent was the premise for completion of the questionnaire. General policy is defined as: ‘the way the large majority of patients (> 75%) with a certain indication would be treated’.

### Statistical analysis

We used descriptive statistics (frequencies and percentages) to present the data. Respondents could indicate how frequently certain management strategies were used (never 0–10%, rarely 10–30%, sometimes 30–70%, frequently 70–90%, and always 90–100%). The combined numbers of respondents that indicated ‘frequently’ and ‘always’ were interpreted as representing the general policy of a center, in line with previous reports [20, 21]. To describe center characteristics in more detail we divided centers into higher (Austria, Belgium, Denmark, Finland, France, Germany, Israel, Italy, the Netherlands, Norway, Spain, Sweden, UK, and Switzerland) versus relatively lower income countries (Bosnia Herzegovina, Hungary, Latvia, Lithuania, Romania, and Serbia), based on a 2007 report by the European Commission [22]. Differences were assessed for statistical significance using the Fisher’s exact test without correction for multiple comparisons. We used Statistical Package for Social Sciences (SPSS) version 21 [23] for descriptive analyses.

## Results

### Participating centers

Of the 68 neurotrauma centers participating in this study, 66 (97%) centers completed the questions on general supportive and preventive ICU management. The questionnaire was predominantly completed by intensivists (*n* = 33, 50%) and neurosurgeons (*n* = 23, 35%). Other professionals that assisted in completion of the questionnaire were administrative staff (*n* = 11, 17%), neurologists (*n* = 5, 8%), anesthesiologists (*n* = 5, 8%), and a trauma surgeon (*n* = 1, 2%).

The majority of centers had an academic affiliation (*n* = 60, 91%). The majority of centers were designated as level I trauma centers (*n* = 45, 69%), and a minority as level II (*n* = 4, 6%), level III (*n* = 1, 2%), or no designation (*n* = 15, 23%). More than half of the centers had a dedicated neuroICU (defined as an ICU that is equipped to treat patients with neurological or neurosurgical injury) available (*n* = 39, 59%). The majority of centers adopted a ‘closed’ ICU organization (the intensivist is primarily responsible for the delivery of care for patients at the ICU) (*n* = 43, 65%), followed by a ‘mixed’ ICU organization (the admitting physician, e.g., neurosurgeon, is primarily responsible but the care is provided by a intensivist) (*n* = 20, 30%), and a minority adopted an ‘open’ ICU organization (the admitting physician is primarily responsible for care at the ICU) (*n* = 3, 5%). Centers indicated to treat a median of 92 (interquartile range 52–160) patients with TBI at their ICU annually. Twenty-eight centers (42%) reported to adhere to the 2007 Brain Trauma Foundation (BTF) guidelines for the management of patients with TBI at their ICU, and 21 centers (32%) reported having institutional guidelines that were based on BTF guidelines. The center characteristics and definitions are described in more detail in a previous publication [19].

### Circulatory and respiratory management

As part of circulatory management, the most frequently mentioned cerebral perfusion pressure (CPP) targets were > 60 mmHg (*n* = 39, 60%) and/or “individualized” (*n* = 25, 38%). Most centers used crystalloids (*n* = 60, 91%) and/or vasopressors (*n* = 63, 96%) for CPP support; inotropes (*n* = 29, 44%) were less frequently, but still regularly, employed. Fifteen centers (23%) reported to use albumin-containing solutions for volume expansion (Additional file 2: Table S1).

**Fig. 1:**
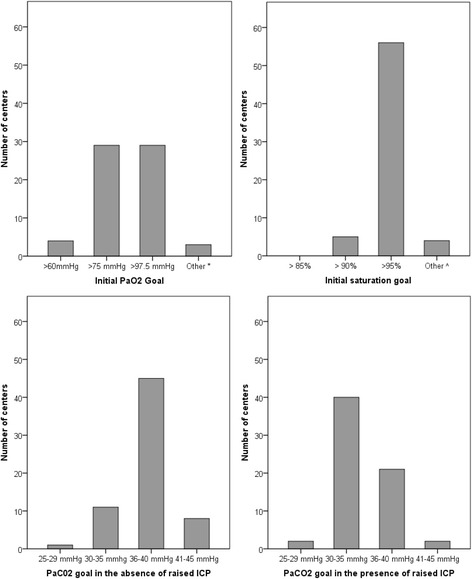
Mechanical ventilation thresholds with corresponding answer frequencies; 25–29 mmHg ≈ 3.3–3.0 kPa, 30–35 mmHg ≈ 4–4.7 kPa, 36–40 mmHg ≈ 4.8–5.3 kPa, 41–45 mmHg ≈ 5.5–6 kPa, 60 mmHg = 8 kPa, 75 mmHg = 10 kPa, 100 mmHg = 13 kPa. * No specific goal (*n* = 1), > 90 mmHg (*n* = 2); ^^^ > 96% (*n* = 2), > 97% (*n* = 1), 92–94% (*n* = 1). PaCO2 partial pressure of carbon dioxide in arterial blood, PaO2 partial pressure of oxygen in arterial blood

In mechanically ventilated patients with TBI, initial partial pressure of oxygen in arterial blood (PaO_2_) goals of > 75 mmHg (10 kPa) (*n* = 29, 45%) and > 97.5 mmHg (13 kPa) (*n* = 29, 45%) were most commonly cited as a treatment preference, with an initial arterial oxygen saturation goal of > 95% (*n* = 56, 86%). In the absence of raised ICP, most centers indicated a partial pressure of carbon dioxide in arterial blood (PaCO_2_) goal of 36–40 mmHg (4.8–5.3 kPa) (*n* = 45, 69%). In the presence of raised ICP this shifted towards a lower PaCO_2_ goal of 30–35 mmHg (4.0–4.7 kPa) (*n* = 40, 62%) (Fig. 1). The timing of tracheostomy in patients with limited or slow neurological recovery varied substantially from within 1 week (*n* = 13, 20%) to between 1 and 2 weeks (*n* = 36, 55%) and more than 2 weeks (*n* = 16, 25%) (Additional file 2: Table S1).

Relatively lower income countries more frequently adopted lower oxygen saturation goals (> 90%) compared with saturation targets > 95% which were favored by higher income countries (*n* = 3/11, 27%, versus *n* = 2/55, 4%; *p* = 0.037) (Additional file 3: Table S6).

### Fever control

**Fig. 2:**
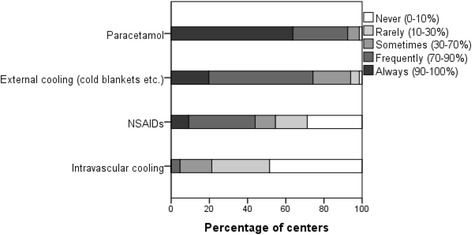
Type of fever treatment and corresponding percentage of centers that indicated they use this type of fever treatment never (in 0–10% of cases), rarely (in 10–30% of cases), sometimes (in 30–70% of cases), frequently (in 70–90% of cases), or always (in 90–100% of cases). NSAID nonsteroidal anti-inflammatory drug

In patients with TBI, the majority of centers indicated that they routinely treat fever (*n* = 65, 98%). One center (2%) reported they would only treat fever “sometimes”. The preferred treatments were paracetamol (*n* = 61, 92%) and/or external cooling (*n* = 49, 74%). In contrast, nonsteroidal anti-inflammatory drugs (NSAIDs) were less commonly used (*n* = 29, 44%). Intravascular cooling was also rarely used (*n* = 3, 5%) (Fig. 2) (Additional file 2: Table S2).

Relatively lower income countries significantly indicated the use of NSAIDs more often than higher income countries (*n* = 11/11, 100%, versus *n* = 18/55, 33%; *p* = 0.000). Centers in higher income countries indicated the use of paracetamol significantly more frequently compared with relatively lower income countries (*n* = 53/55, 96%, versus *n* = 8/11, 73%; *p* = 0.029). Intravascular cooling was more frequently applied in the lower income group, although this difference did not reach statistical significance (Additional file 3: Table S7).

### Use of corticosteroids

Corticosteroids were infrequently used for the primary management of brain injury, although a few respondents indicated that they used them “rarely” (*n* = 5, 8%), “sometimes” (*n* = 2, 3%), or “frequently” (*n* = 1, 2%). However, corticosteroids were specifically used for vasopressor-resistant hypotension (*n* = 21, 58%) and, to a lesser extent, sepsis (*n* = 8, 22%) (Additional file 2: Table S3).

Primary use of corticosteroids was significantly more frequently reported by lower income countries compared with higher income countries (*n* = 4/11, 36%, versus *n* = 4/55, 7%; *p* = 0.023) (Additional file 3: Table S7).

### Glucose and nutrition management

The majority of centers stated that their glucose management was protocolized (*n* = 50, 77%). Most centers reported the correction of hyperglycemia as a primary aim (*n* = 43, 66%) while a smaller number implemented tight glycemic control (*n* = 18, 28%) (Additional file 2: Table S4).

Most respondents aimed for full caloric replacement within 7 days post-injury (*n* = 43, 66%). An open question on the goals for caloric intake showed a high variety in reported strategies as well as metrics used (kcal/day, kcal/kg/day, and percentages). The enteral route was preferred (*n* = 60, 92%). The timing of parenteral nutrition was highly variable: centers were equally distributed between “as soon as possible” (*n* = 13, 20%), “within 24 h post-injury” (*n* = 13, 20%), “within 72 h post-injury” (*n* = 10, 15%), “within 7 days post-injury” (*n* = 17, 26%), and “we do not have rules/guidelines for this” (*n* = 12, 19%) (Additional file 2: Table S4).

Relatively lower income countries reported using the parenteral route significantly more frequently compared with higher income countries (*n* = 4/11, 36%, versus *n* = 1/55, 2%, *p* = 0.002) (Additional file 3: Table S7).

### Seizure prophylaxis and treatment

**Fig. 3:**
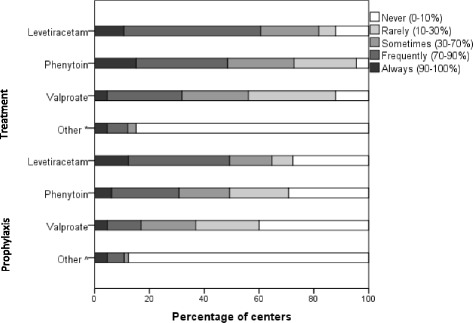
Agents for seizure prophylaxis and treatment with corresponding percentage of centers that indicated that they never (in 0–10% of cases), rarely (in 10–30% of cases), sometimes (in 30–70% of cases), frequently (in 70–90% of cases), or always (in 90–100% of cases) use the agent. *Carbamazepine/phenobarbital, phenobarbital, benzodiazepines, no prophylaxis used in our hospital, carbamazepine (*n* = 3). ^^^Phenobarbital, benzodiazepines, carbamazepine (*n* = 4), midazolam/diazepam, lorazepam

There was little consensus regarding the use of prophylactic antiepileptic drugs (for all indications). Most centers reported to use levetiracetam as the drug of choice for both seizure prophylaxis and treatment (*n* = 32, 49%, and *n* = 40, 61%), followed by phenytoin (*n* = 20, 31%, and *n* = 32, 48%) (Fig. 3). In general, both the reported duration of antiseizure prophylaxis and the criteria for initiation of antiepileptic treatment varied considerably (Additional file 2: Table S5).

The choice of agent varied with income, with levetiracetam being less commonly used for both seizure prophylaxis (*n* = 0/11 versus *n* = 32/55, 59%; *p* = 0.000) and treatment (*n* = 1/11, 9%, versus *n* = 39/55, 71%; *p* = 0.000) in the lower income group versus higher income countries, respectively. Instead, lower income countries seemed to favor valproate or phenytoin compared with higher income countries (*n* = 7/11, 64%, versus *n* = 14/55, 26%; *p* = 0.029) (Additional file 3: Table S7).

## Discussion

In this survey, we found varying degrees of consensus between European neurotrauma centers with respect to general supportive and preventive ICU management in patients with TBI. Most variation was found in initial PaO_2_ goals for mechanically ventilated patients, CPP targets, the timing of tracheostomy in unconscious patients, nutritional targets, and seizure prophylaxis and treatment.

Large between-center variation was found in topics that are not addressed in the recommendations of the BTF guidelines (Additional file 4), suggesting the role of guidelines in reducing variances in clinical practice. International guidelines (BTF guidelines and guidelines of the American College of Surgeons) do recommend the use of normalized thresholds (e.g., normoglycemia, normocapnia, and normothermia) in patients with TBI, although this is not based on high-level evidence [16, 17]. Indeed, randomized controlled trials (RCTs) on these topics are too limited in number to lead to high-level evidence [10]. Considering CPP targets, the BTF guidelines are unclear whether to use an optimum threshold of > 60 or > 70 mmHg (and a range of 50–70 mmHg in the previous BTF guidelines [24]). Despite this ambiguity, a majority of respondents (60%) preferred a target CPP of > 60 mmHg. In addition, the current BTF guidelines added that the CPP target might depend on the individual cerebral autoregulatory status, reflected by 38% of respondents who indicated to use an individualized target CPP. The uniformity in reported CPP targets between income groups also suggests that these concepts are widespread. It may be that the willingness to individualize CPP in patients with TBI reflects the growing trend for use of precision medicine [25], where therapies and therapy targets are individualized to patient need, rather than used on a “one size fits all” basis.

Marked variation was also found on topics where consensus was expected based on high-level evidence from RCTs or the recommendations in the BTF guidelines. The use of steroids for the primary management of TBI was reported by 13% of the respondents (one respondent reported frequent use), but is against the advice of the BTF guidelines and contradicts the prevailing evidence from the CRASH study [26, 27]. However, use in the majority of centers was for vasopressor dependence and/or sepsis, a use in keeping with current guidelines for the management of sepsis [28]. The use of albumin was reported by 23% of the respondents, while the SAFE study showed that albumin was associated with higher mortality rates in patients with TBI [29]. It is difficult to interpret the continued use of albumin for volume expansion as a lack of knowledge of the evidence, since worse outcomes in the albumin-treated arm in SAFE-TBI may have been the consequence of a hypotonic carrier causing elevated ICP [30], and well-informed clinicians may have used albumin that was isotonic or corrected any accompanying hyponatremia. Finally, the use of tight glycemic control was reported by 28% of respondents, while the NICE-SUGAR and CGAO-REA studies recommend using moderate instead of tight glucose control in patients with TBI [31, 32].

On the other hand, we found consensus where variation was expected; a high number of centers indicated they use antipyretic agents for the treatment of fever when there is no consensus on the optimal choice of agent and when their potentially deleterious side-effect of CPP lowering is well known [33]. This suggests a strong aversion amongst treating clinicians to allow pyrexia in patients with TBI. The choice of NSAIDs, despite their well-known potentially harmful systemic side-effect profile, as antipyretics in many centers probably also reflects this, although a continuous intravenous infusion instead of intermittent NSAID dosing might improve fever control (with relatively higher CPP) in neurocritical care [34]. In addition, respondents indicated employing below-normal PaCO_2_ goals (30–35 mmHg) in the presence of raised ICP in mechanically ventilated TBI patients. This was unexpected given the BTF recommendation to avoid prolonged hyperventilation. Furthermore, even patients in whom intracranial hypertension was not a concern were ventilated to normal carbon dioxide tensions showing a reluctance to use permissive ventilatory strategies that have been shown to be effective in reducing mortality in acute respiratory distress syndrome (ARDS) patients [35].

Our results further suggest that respondents use TBI-specific strategies instead of general strategies (as used in the general critically ill patients) in the ICU. For example, respondents indicated they frequently or always treat fever since hyperthermia is associated with worse outcomes in TBI [14, 33], whereas fever is often considered beneficial to some extent in critically ill patients with infections [36].

We found some differences between relatively lower versus higher income countries. It was striking that levetiracetam was significantly more frequently reported by higher income countries as an agent of choice for seizure prophylaxis and treatment, while valproate and phenytoin were reported more frequently by lower income countries, although high-level evidence in the literature on the agent of choice is lacking [37]. However, there were no clear structural differences in management overall, and this could not therefore be considered an explanation for the treatment variation. Indeed, some high-cost interventions, such as intravascular cooling and parenteral nutrition, were more commonly used in the lower income countries, suggesting that choices of treatment options are not solely based on cost considerations, but also reflect local clinical culture in different institutions.

Our study has several strengths. To our knowledge, this is the first survey that provides an overview of multiple components of general supportive or preventive ICU management in patients with TBI. The survey was developed in several stages with involvement of clinical experts of various disciplines and the response rate of the survey was high (97%). However, this study also has limitations, as the centers participating in the CENTER-TBI study may still be a biased selection of European centers with a specialist interest in the topic, or a large engagement in research, or more expertise overall. In a small number of centers, the questionnaire was completed by administrative staff (with no clinical expertise). However, presumably this was in close collaboration with a clinician considering the high number of clinicians that completed the survey, and clinical involvement was encouraged throughout the survey. Other limitations are inherent to surveys in that the results are self-reported and are not confirmed by independent observations in daily practice and, therefore, represent what the respondents ‘believe’ is clinical practice and this may not, in fact, reflect reality. Another limitation is that the survey questions represent generalizations and do not include patient factors (such as demographics, laboratory results, or imaging), or very specific circumstances, while in clinical practice these details influence clinicians’ judgement. In line with this, we did not specify time frames (for ventilation goals) and laboratory values (for tight glucose control). Also, we asked about general patients with TBI in the survey and did not specify adult or pediatric TBI.

Overall, the practice variation (and consensus) in general ICU management we found might be explained by a lack of evidence (or incomplete implementation of evidence), by the use of individualized approaches, or by a tension between general and TBI-specific strategies. We presume that increased and more evidence-based uniformity in good practices in general ICU management might improve outcome in TBI. In fact, general ICU management is part of daily routine (e.g., temperature measurements, laboratory results, and mechanical ventilation) and deviations are generally easily detected and corrected. It is noteworthy that non-neurological complications are frequent; in one report on TBI patients these were more frequent (around 22%) than neurological complications (around 3%) [29]. Our survey showed that future research on individualized management is needed; a high number of respondents reported individualized practices which implies a trend towards precision medicine. In addition, the existence of practice variation in general ICU management provides direction to comparative effectiveness research (CER) analyses or RCTs. As RCTs in the field of TBI have been disappointing [10], CER might be a promising approach to enhance future knowledge on the effectiveness of general ICU management, and understanding what process variances occur, as we have attempted to do, is a critical starting point. Hence, in the CENTER-TBI study we will evaluate the effect of different ICU management practices on TBI outcome (after case-mix correction); for example, the difference in patient outcome between the 13 centers that plan tracheostomy within 1 week, the 36 centers that time tracheostomy between 1 and 2 weeks, and the 16 centers that delay tracheostomy longer than 2 weeks.

## Conclusions

This study shows that general supportive and preventive ICU management policies in TBI vary between European neurotrauma centers. These findings stress the need for continued knowledge transfer of existing evidence, further research on optimized individualized management (precision medicine) and, as we propose, comparative effectiveness research.

## Additional files

Additional file 1: Survey questions: survey questions of the ‘Provider Profiling Questionnaire’ used in the study (treatment in the intensive care unit). (DOCX 23 kb)

Additional file 2: Overview of all results: circulatory and respiratory management (**Table S1**), fever control (**Table S2**), use of corticosteroids (**Table S3**), glucose and nutrition management (**Table S4**), seizure prophylaxis and treatment (**Table S5**). (DOCX 26 kb)

Additional file 3: Variation between higher and lower income countries: variation in thresholds used for circulatory and respiratory management (**Table S6**) and general treatments in the ICU (**Table S7**). (DOCX 22 kb)

Additional file 4: Comparison with the Brain Trauma foundation recommendations: items of the questionnaire with corresponding recommendations in the Brain Trauma Foundation guidelines for the Management of Severe Traumatic Brain Injury (4th edition). (DOCX 17 kb)

Additional file 5: CENTER-TBI investigators and participants participating in the CENTER-TBI study and their corresponding affiliations. (DOCX 33 kb)

## Abbreviations

BTF: Brain trauma foundation
CENTER-TBI: Collaborative european neurotrauma effectiveness research in traumatic brain injury
CER: Comparative effectiveness research
CPP: Cerebral perfusion pressure
ICP: Intracranial pressure
ICU: Intensive care unit
NSAID: Nonsteroidal anti-inflammatory drug
PaCO_2_: Partial pressure of carbon dioxide in arterial blood
PaO_2_: Partial pressure of oxygen in arterial blood
RCT: Randomized controlled trial
TBI: Traumatic brain injury
