# Supplementary material for: Variation in general supportive and preventive intensive care management of traumatic brain injury: a survey in 66 neurotrauma centers participating in the Collaborative European NeuroTrauma Effectiveness Research in Traumatic Brain Injury (CENTER-TBI) study
Source: Crit Care. 2018 Apr 13;22:90. doi: 10.1186/s13054-018-2000-6 (PMC5898014; doi:10.1186/s13054-018-2000-6)
Supplement: Supplementary file 1 — Survey questions: survey questions of the ‘Provider Profiling Questionnaire’ used in the study (treatment in the intensive care unit). (DOCX 23 kb) [file 13054_2018_2000_MOESM1_ESM.docx]

Survey questions: treatment at the Intensive Care Unit (additional file 1)

**Information about the completer of the questionnaire**

Other than the CENTER-TBI investigator, which of the following individuals was involved in completion of this questionnaire?

*Select all that apply*

- Neurologist
- Neurosurgeon
- Trauma Surgeon
- ED physician
- Administrative staff member / data manager / financial department
- Other, please specify……………..
- NA. The questionnaire is solely completed by the CENTER TBI local investigator

The Local investigator is the senior clinician(s) at your hospital involved in supervision of CENTER TBI

**General patient statistics**

What is the number of Traumatic Brain Injury (TBI) patients treated in your Intensive Care Unit (ICU) annually?

1. 2012: ……………………………………..
2. 2013: ……………………………………..

***CPP monitoring***

30. Please list the target Cerebral Perfusion Pressure utilized at your facility:

*Select all that apply*

- > 50 mmHg
- > 60 mmHg
- > 70 mmHg
- Individualized

31. For treating CPP, which types of IV fluids are used to augment intravascular volume?

*Select all that apply*

- Crystalloids
- Colloids – starches
- Colloids - albumin
- Other combinations

32. Which vasoactive drugs are used to support CPP in patients with Traumatic Brain Injury (TBI)?

*Select all that apply*

- Vasopressors
- Inotropes
- Other, please specify………

***Seizure prophylaxis and management***

The responses to the following questions should represent, as best as practicable, a general consensus on treatment at your centre, rather than individual management preferences.  These questions refer to the use of drugs to prevent seizures, rather than treat documented seizures

44. What are indications for anti-seizure prophylaxis in your centre?

|  | Never (0-10%) | Rarely (10-30%) | Sometimes (30-70%) | Frequently (70-90%) | Always (90-100%) |
| --- | --- | --- | --- | --- | --- |
| Glasgow Coma Scale (GCS) < 10 |  |  |  |  |  |
| Cortical contusion |  |  |  |  |  |
| Depressed skull fracture |  |  |  |  |  |
| Subdural hematoma |  |  |  |  |  |
| Epidural hematoma |  |  |  |  |  |
| Intracerebral hematoma |  |  |  |  |  |
| Penetrating brain injury |  |  |  |  |  |
| Other – please specify  ………………………………..  ………………………………… |  |  |  |  |  |

45. What is the duration of recommended anti-seizure prophylaxis at your hospital?

*Select all that apply*

- 1-3 days
- 4-7 days
- >7 days
- 3 weeks
- 3 months
- Variable depending on patient
- Variable depending on physician

46. Please rate the utilization of following agents used for seizure prophylaxis of Traumatic Brain Injury (TBI) patients:

|  | Always (90-100%) | Frequently (90-70%) | Sometimes (30-70%) | Rarely (10-30%) | Never (0-10%) |
| --- | --- | --- | --- | --- | --- |
| Phenytoin |  |  |  |  |  |
| Levetiracetam |  |  |  |  |  |
| Valproate |  |  |  |  |  |
| Other, please specify……………….. |  |  |  |  |  |

***Treatment of seizures***

The responses to the following questions should represent, as best as practicable, a general consensus on treatment at your centre, rather than individual management preferences.

47. Does your Intensive Care Unit (ICU) initiate anti-epileptic treatment after:

|  | Never (0-10%) | Rarely (10-30%) | Sometimes (30-70%) | Frequently (70-90%) | Always (90-100%) |
| --- | --- | --- | --- | --- | --- |
| A single seizure |  |  |  |  |  |
| Two or more seizures |  |  |  |  |  |

48. Please rate your utilization of following agents used for seizure treatment of Traumatic Brain Injury (TBI) patients:

|  | Never (0-10%) | Rarely (10-30%) | Sometimes (30-70%) | Frequently (70-90%) | Always (90-100%) |
| --- | --- | --- | --- | --- | --- |
| Phenytoin |  |  |  |  |  |
| Levetiracetam |  |  |  |  |  |
| Valproate |  |  |  |  |  |
| Other, please specify……………….. |  |  |  |  |  |

***Fever***

The responses to the following questions should represent, as best as practicable, a general consensus on treatment at your centre, rather than individual management preferences.

49. Is fever (core temperature >38) in Traumatic Brain Injury (TBI) patients routinely treated in your unit?

- Never (0-10%)
- Rarely (10-30%)
- Sometimes (30-70%)
- Frequently (70-90%)
- Always (90-100%)

50. Please rate your utilization of the following approaches for management of fever in Traumatic Brain Injury (TBI) patients with increased ICP:

|  | Never (0-10%) | Rarely (10-30%) | Sometimes (30-70%) | Frequently (70-90%) | Always (90-100%) |
| --- | --- | --- | --- | --- | --- |
| Paracetamol |  |  |  |  |  |
| NSAIDs |  |  |  |  |  |
| External cooling (cold blankets, etc) |  |  |  |  |  |
| Intravascular cooling |  |  |  |  |  |

|  | Never (0-10%) | Rarely (10-30%) | Sometimes (30-70%) | Frequently (70-90%) | Always (90-100%) |
| --- | --- | --- | --- | --- | --- |
| 51. Are corticosteroids used for the primary management of TBI in patients with head injury? |  |  |  |  |  |
| 52. Are corticosteroids used for ICU management of other conditions in TBI patients (e.g.: vasopressor resistant hypotension)?  Specify: ……………………………….. |  |  |  |  |  |

**Respiration and ventilation**

The responses to the following questions should represent, as best as practicable, a general consensus on treatment at your centre, rather than individual management preferences.

**Mechanical ventilation**

57. Select initial PaO2 goal in mechanically ventilated Traumatic Brain Injury (TBI) patients:

- > 8 kpa (60 mmHg)
- > 10 kpha (75mmHg)
- >13 kpa (100 mmHg)
- Other, please specify……………………

58. Select initial arterial oxygen saturation goal in mechanically ventilated Traumatic Brain Injury (TBI) patients:

- > 85%
- > 90 %
- > 95%
- Other, please specify……………………………

59. In patients remaining unconscious after Traumatic Brain Injury (TBI) , when would your ICU consider a tracheostomy?

- Within 1 week
- 1- 2 weeks
- > 2weeks

60. Select PaCO2 goal for management in mechanically ventilated Traumatic Brain Injury (TBI) patients:

|  | 25-29 mmHg | 30-35 mmHg | 36-40 mmHg | 41-45 mmHg |
| --- | --- | --- | --- | --- |
| In the absence of (suspicion of) raised ICP |  |  |  |  |
| In the presence of raised ICP |  |  |  |  |

***Glucose***

64. Is there a standard protocol for glucose management in Traumatic Brain Injury (TBI) in your Intensive Care Unit (ICU) ?

- No
- Yes

65. What therapy is used in glucose management at your Intensive Care Unit (ICU) ?

- No specific therapy
- Prophylactic insulin administration (buffered infusion)
- Insulin administration to correct hyperglycemias
- Tight glycemic control

***Caloric intake / nutrition***

66. How is nutrition managed at your Intensive Care Unit (ICU) ?

- Always parenteral route
- Always Enteral route
- Mostly parenteral route, enteral route on indication
- Mostly enteral route, parenteral route on indication
- Other………………………………………….

67. What caloric intake do you aim for in patients with Traumatic Brain Injury (TBI) at your Intensive Care Unit (ICU) ?

………………………. Kcal/kg/day

- Unknown / no protocol

68. When do you usually start parenteral nutrition at your Intensive Care Unit (ICU) in Traumatic Brain Injury (TBI) patients?

- As soon as possible (directly after ICU admission)
- Within 24 hours post-injury
- Within 72 hours post-injury
- Within 7 days post-injury
- We do not have rules / guidelines for this

69. When do you usually aim for full caloric intake replacement?

- At 7 days post-injury
- < 7 days post-injury
- > 7 days post-injury
