# Supplementary material for: Variation in general supportive and preventive intensive care management of traumatic brain injury: a survey in 66 neurotrauma centers participating in the Collaborative European NeuroTrauma Effectiveness Research in Traumatic Brain Injury (CENTER-TBI) study
Source: Crit Care. 2018 Apr 13;22:90. doi: 10.1186/s13054-018-2000-6 (PMC5898014; doi:10.1186/s13054-018-2000-6)
Supplement: Supplementary file 2 — Overview of all results: circulatory and respiratory management (Table S1), fever control (Table S2), use of corticosteroids (Table S3), glucose and nutrition management (Table S4), seizure prophylaxis and treatment (Table S5). (DOCX 26 kb) [file 13054_2018_2000_MOESM2_ESM.docx]

Overview of all results (additional file 2)

| Table 1. Circulatory and respiratory management | | | |
| --- | --- | --- | --- |
| Items of the questionnaire | Number completed | N (%) | |
| Circulatory management |  |  | |
| Target CPP   - >50 mmHg - >60 mmHg - >70 mmHg - Individualized | 66* | 7  39  14  25 | 11  60  21  38 |
| IV fluids for treatment CPP   - Crystalloids - Colloids - starches - Colloids - albumin - Other combinations | 66* | 60  10  15  8 | 91  15  23  12 |
| Vasoactive drugs to support CPP   - Vasopressors - Inotropes | 66* | 63  29 | 96  44 |
| Respiratory management |  |  |  |
| Initial PaO_2_ goal ^1^   - > 8 kPa (60 mmHg) - > 10 kPa (75 mmHg) - >13 kPa (100 mmHg) - Other ^2^ | 65 | 4  29  29  3 | 6  45  45  4 |
| Initial arterial oxygen saturation goal   - >85% - >90% - >95% - Other ^3^ | 65 | 0  5  56  4 | 0  8  86  6 |
| PaCO_2_ goal - in the absence of raised ICP   - 25-29 mmHg (≈ 3.3-3.0 kPa) - 30-35 mmHg (≈ 4-4.7 kPa) - 36-40 mmHg (≈ 4.8-5.3 kPa) - 41-45 mmHg (≈ 5.5-6 kPa) | 65 | 1  11  45  8 | 2  17  69  12 |
| PaCO_2_ goal - in the presence of raised ICP   - 25-29 mmHg (≈ 3.3-3.0 kPa) - 30-35 mmHg (≈ 4-4.7 kPa) - 36-40 mmHg (≈ 4.8-5.3 kPa) - 41-45 mmHg (≈ 5.5-6 kPa) | 65 | 2  40  21  2 | 3  62  32  3 |
| Timing tracheotomy ^4^   - < 1 week - 1-2 weeks - >2 weeks | 65 | 13  36  16 | 20  55  25 |
| 1) In mechanically ventilated patients, 2) No specific goal (N=1), > 12 kPa (N=2), 3) > 96% (N=2), >97% (N=1), 92-94% (N=1), 4) In patients remaining unconscious  * Multiple answers were possible  CPP: Cerebral Perfusion Pressure, ICP: intracranial pressure, IV: intravenous, mmHg: millimeters of mercury, PaCO_2_: partial pressure of carbon dioxide in arterial blood, PaO_2_: partial pressure of oxygen in arterial blood | | | |

| Table 2. Fever control | | | |
| --- | --- | --- | --- |
| Items of the questionnaire | Number completed | N (%) | |
| Fever ^1^ treated routinely   - Never (0-10%) - Rarely (10-30%) - Sometimes (30-70%) - Frequently (70-90%) - Always (90-100%) | 66 | 0  0  1  21  44 | 0  0  2  32  66 |
| Type of treatment of fever   - Paracetamol - NSAIDs - External cooling ^2^ - Intravascular cooling | 66*, ** | 61  29  49  3 | 92  44  74  5 |
| 1) Core temperature above 38 °C 2) Cold blankets etc  * Multiple answers were possible  ** Sum of centers that indicated frequently (70-90% of cases) or always (100% of cases)  NSAIDs: nonsteroidal anti-inflammatory drugs | | | |

| Table 3. Use of corticosteroids |  |  |  |
| --- | --- | --- | --- |
| Items of the questionnaire | Number completed | N | (%) |
| Primary management with corticosteroids   - Never (0-10%) - Rarely (10-30% - Sometimes (30-70%) - Frequently (70-90%) - Always (90-100%) | 66 | 57  5  2  1  0 | 87  8  3  2  0 |
| Corticosteroids used for other conditions (open question)   - Vasopressor resistant hypotension - Sepsis - Other ^1^ | 36** | 21  8  7 | 58  22  20 |
| 1) Adrenal insufficiency, bronchospasm, spinal cord injury, hypopituitarism, hypocortisolism, spinal cord injury, peripheric nerve injury, stress response  * Multiple answers were possible  ** Sum of centers that indicated frequently (70-90% of cases) or always (100% of cases) | | | |

| Table 4. Glucose and nutrition management | | | |
| --- | --- | --- | --- |
| Items of the questionnaire | Number completed | N (%) | |
| Glucose management |  |  | |
| Protocol glucose management   - Presence of a protocol - Absence of a protocol | 65 | 50  15 | 77  23 |
| Glucose therapy   - No specific therapy - Prophylactic insulin administration^1^ - Insulin administration to correct hyperglycemias - Tight glycemic control | 65 | 2  2  43  18 | 3  3  66  28 |
| Nutrition management |  |  |  |
| Aim for full caloric replacement   - At 7 days post-injury - < 7 days post-injury - >7 days post-injury | 65 | 12  43  10 | 19  66  15 |
| Aim caloric intake (open question)   - 1900 kcal/day^2^ - 27 kcal/kg/day^3^ - Other ^4^ - Unknown/no protocol | 65 | 14  32  10  9 | 22  49  15  14 |
| Route of nutrition   - Parenteral - Enteral^5^ | 65 | 5  60 | 8  92 |
| Start parenteral nutrition   - As soon as possible ^6^ - Within 24 hours post-injury - Within 72 hours post-injury - Within 7 days post-injury - We do not have rules/ guidelines for this | 65 | 13  13  10  17  12 | 20  20  15  26  19 |
| 1) Buffered infusion; 2) median 3) median 4) 100%-130%; 80%ee; 100%; 2 kcal/kg/h; for patients with no resp; variable; based on calorimetry; high 5) Including mostly enteral, parenteral on indication 6) Directly after ICU admission  kcal: kilocalories, kg: kilograms | | | |

| Table 5. Seizure prophylaxis and treatment | | | |
| --- | --- | --- | --- |
| Items of the questionnaire | Number completed | N (%) | |
| General |  |  | |
| Indications for anti-seizure prophylaxis   - GCS<10 - Cortical contusion - Depressed skull fracture - Subdural hematoma - Epidural hematoma - Intracerebral hematoma - Penetrating brain injury - Other ^1^ | 66* , ** | 15  21  23  19  12  19  25  5 | 23  32  35  29  18  29  38  8 |
| Seizure prophylaxis |  |  |  |
| Agents used for seizure prophylaxis   - Phenytoin - Levetiracetam - Valproate - Other ^2^ | 65*, ** | 20  32  11  7 | 31  49  17  11 |
| Duration of anti-seizure prophylaxis   - 1-3 days - 4-7 days - > 7 days - 3 weeks - 3 months - Depending on the patient - Depending on the physician | 66* | 3  21  6  2  5  22  12 | 5  32  9  3  8  33  18 |
| Seizure treatment |  |  |  |
| Agents used for seizure treatment   - Phenytoin - Levetiracetam - Valproate - Other ^3^ | 66*, ** | 32  40  21  8 | 48  61  32  12 |
| Initiation of anti-epileptic treatment   - A single seizure - Two or more seizures | 66** | 44  61 | 67  92 |
| 1) Previous seizure, seizure within 24 hours, epileptic fit, ventilated TBI, all severe, EEG confirmed seizures, 2) Carbamazepine/ phenobarbital, phenobarbital, benzodiazepines, no prophylaxis used in our hospital, carbamazepine (N=3), 3) Phenobarbital, benzodiazepines, carbamazepine (N=4), midazolam/diazepam, lorazepam  * Multiple answers were possible  ** Sum of centers that indicated frequently (70-90% of cases) or always (100% of cases)  GCS: Glasgow Coma Scale | | | |
