# Supplementary material for: Variation in general supportive and preventive intensive care management of traumatic brain injury: a survey in 66 neurotrauma centers participating in the Collaborative European NeuroTrauma Effectiveness Research in Traumatic Brain Injury (CENTER-TBI) study
Source: Crit Care. 2018 Apr 13;22:90. doi: 10.1186/s13054-018-2000-6 (PMC5898014; doi:10.1186/s13054-018-2000-6)
Supplement: Supplementary file 3 — Variation between higher and lower income countries: variation in thresholds used for circulatory and respiratory management (Table S6) and general treatments in the ICU (Table S7). (DOCX 22 kb) [file 13054_2018_2000_MOESM3_ESM.docx]

Variation between higher and lower income countries (additional file 3)

| Table 1. Thresholds used for circulatory and respiratory management | | | | | | |
| --- | --- | --- | --- | --- | --- | --- |
| Items of the questionnaire | Higher income countries  (N=55) | | Lower income countries (N=11) | | | P-value |
| Respiratory management |  |  | |  |  | |
| Initial PaO_2_ goal ( in mechanically ventilated patients)   - > 10 kPa (75 mmHg) - >13 kPa (100 mmHg) | 25 (50%)  25 (50%) | | 4 (50%)  4 (50%) | | | 1.000 |
| Initial arterial oxygen saturation goal   - >90% - >95% | 2 (4%)  48 (96%) | | 3 (27%)  8 (73%) | | | **0.037** |
| PaCO_2_ goal - in the absence of raised ICP   - 25-35mmHg - 36-45 mmHg | 8 (15%)  46 (85%) | | 4 (36%)  7 (64%) | | | 0.194 |
| PaCO_2_ goal - in the presence of raised ICP   - 25-35 mmHg - 36-45 mmHg | 35 (65%)  19 (35%) | | 7 (64%)  4 (36%) | | | 1.000 |
| Circulatory management |  |  | |  |  | |
| Target CPP   - >50 mmHg - >60 mmHg - >70 mmHg - Individualized | 7 (13%)  33 (60%)  13 (24%)  20 (36%) | | 0 (0%)  6 (55%)  1 (9%)  5 (45%) | | | 0.591  0.749  0.433  0.735 |
| In order to calculate the Fisher exact test a sufficient number per category (answer option) was needed, therefore categories with low numbers were deleted (for PaO_2_ goal and saturation goal) or categories were combined (for PaCO_2_ goal in the presence and absence of raised ICP)  *Higher income:* Austria, Belgium, Denmark, Finland, France, Germany, Israel, Italy, the Netherlands, Norway, Spain, Sweden, the UK and Switzerland; *Relatively low income:* Bosnia Herzegovina, Hungary, Latvia, Lithuania, Romania and Serbia.  CPP: Cerebral Perfusion Pressure, ICP: intracranial pressure, IV: intravenous, mmHg: millimeters of mercury, PaCO_2_: partial pressure of carbon dioxide in arterial blood, PaO_2_: partial pressure of oxygen in arterial blood | | | | | | |

| Table 2. General treatments at the ICU | | | |
| --- | --- | --- | --- |
| Items of the questionnaire | Higher income countries (N=55) | Lower income countries (N=11) | P-value |
| Respiratory and circulatory management | |  |  |
| IV fluids   - Crystalloids - Colloids- starches - Colloids- albumin - Other combinations | 51 (93%)  6 (11%)  12 (22%)  5 (9%) | 9 (82%)  4 (36%)  3 (27%)  3 (27%) | 0.260  0.054  0.703  0.122 |
| Vasoactive drugs to support CPP   - Vasopressors - Inotropes | 52 (95%)  24 (44%) | 11 (100%)  5 (46%) | 1.000  1.000 |
| Fever control |  |  |  |
| Type of treatment of fever (general policy)   - Paracetamol - NSAIDs - External cooling - Intravascular cooling | 53 (96%)  18 (33%)  40 (73%)  1 (2%) | 8 (73%)  11 (100%)  9 (82%)  2 (18%) | **0.029**  **0.000**  0.714  0.070 |
| Corticosteroid use |  |  |  |
| Primary management with corticosteroids   - No - Yes | 50 (93%)  4 (7%) | 7 (64%)  4 (36%) | **0.023** |
| Glucose and nutrition management | | | |
| Glucose therapy   - Insulin administration to correct hyperglycemias - Tight glycemic control | 36 (69%)  16 (31%) | 7 (78%)  2 (22%) | 0.713 |
| Route of nutrition   - Parenteral - Enteral | 1 (2%)  53 (98%) | 4 (36%)  7 (64%) | **0.002** |
| Seizure prophylaxis and treatment | | | |
| Agents used for seizure prophylaxis (general policy)   - Phenytoin - Levetiracetam - Valproate | 16 (29%)  32 (59%)  7 (13%) | 4 (36%)  0  4 (36%) | 0.725  **0.000**  0.080 |
| Agents used for seizure treatment (general policy)   - Phenytoin - Levetiracetam - Valproate | 25 (46%)  39 (71%)  14 (26%) | 7 (64%)  1 (9%)  7 (64%) | 0.333  **0.000**  **0.029** |
| In order to calculate the Fisher exact test a sufficient number per category (answer option) was needed, therefore categories were combined (for the primary management with corticosteroids)  *Higher income:* Austria, Belgium, Denmark, Finland, France, Germany, Israel, Italy, the Netherlands, Norway, Spain, Sweden, the UK and Switzerland; *Relatively low income:* Bosnia Herzegovina, Hungary, Latvia, Lithuania, Romania and Serbia.    CPP: cerebral perfusion pressure, IV: intravenous, NSAIDs: nonsteroidal anti-inflammatory drugs | | | |
