# Supplementary material for: Variation in general supportive and preventive intensive care management of traumatic brain injury: a survey in 66 neurotrauma centers participating in the Collaborative European NeuroTrauma Effectiveness Research in Traumatic Brain Injury (CENTER-TBI) study
Source: Crit Care. 2018 Apr 13;22:90. doi: 10.1186/s13054-018-2000-6 (PMC5898014; doi:10.1186/s13054-018-2000-6)
Supplement: Supplementary file 4 — Comparison with the Brain Trauma foundation recommendations: items of the questionnaire with corresponding recommendations in the Brain Trauma Foundation guidelines for the Management of Severe Traumatic Brain Injury (4th edition). (DOCX 17 kb) [file 13054_2018_2000_MOESM4_ESM.docx]

Comparison with the Brain Trauma Foundation (BTF) guidelines recommendations (additional file 4)

| **Items questionnaire Recommendations** | |
| --- | --- |
| **Respiratory and circulatory management** | |
| PaCO_2_ goal (in the presence and absence of raised ICP) | Prolonged prophylactic hyperventilation with partial pressure of carbon dioxide in arterial blood (PaCO2) of 25 mm Hg or less is not recommended. Level II B (Chapter 5. Ventilation Therapies)  Hyperventilation is recommended as a temporizing measure for the reduction of elevated ICP. (Carney et al.)  Hyperventilation should be avoided during the first 24 h after injury when CBF often is reduced critically. (Carney et al.) |
| Initial PaO_2_ goal, arterial saturation goal, timing tracheotomy | - |
| Target CPP | The recommended target cerebral perfusion pressure (CPP) value for survival and favorable outcomes is between 60 and 70 mm Hg. Whether 60 or 70 mm Hg is the minimum optimal CPP threshold is unclear and may depend upon the patient’s autoregulatory status. Level II B (Chapter 13. Cerebral Perfusion Pressure Monitoring) |
| Intravenous fluids (crystolloids, colloids etc.) and vasoactive drugs (inotropes, vasopressors) to support CPP | Avoiding aggressive attempts to maintain CPP above 70 mm Hg with fluids and pressors may be considered because of the risk of adult respiratory failure. Level II (Chapter 13. Cerebral Perfusion Pressure Monitoring) |
| **Fever control** | |
| Type of treatment for fever (pharmaceutical, internal or external cooling) | - |
| **Use of corticosteroids** |  |
| Primary management with corticosteroids | The use of steroids is not recommended for improving outcome or reducing ICP. In patients with severe TBI, high-dose methylprednisolone was associated with increased mortality and is contraindicated. Level I (Chapter 7. Steroids) |
| **Glucose and nutrition management** | |
| Aim and timing for full caloric replacement | Feeding patients to attain basal caloric replacement at least by the fifth day and, at most, by the seventh day post-injury is recommended to decrease mortality. Level II A (Chapter 8. Nutrition) |
| Route of nutrition (parenteral or enteral) | Transgastric jejunal feeding is recommended to reduce the incidence of ventilator-associated pneumonia. Level II B (Chapter 8. Nutrition) |
| Glucose therapy, aim caloric intake, start parenteral nutrition | - |
| **Seizure prophylaxis and treatment** | |
| Agents used for seizure prophylaxis | Prophylactic use of phenytoin or valproate is not recommended for preventing late PTS.  Phenytoin is recommended to decrease the incidence of early PTS (within 7 days of injury), when the overall benefit is felt to outweigh the complications associated with such treatment. However, early PTS have not been associated with worse outcomes. Level II A (Chapter 11. Seizure prophylaxis)  At the present time there is insufficient evidence to recommend levetiracetam compared with phenytoin regarding efficacy in preventing early post-traumatic seizures and toxicity. (Carney et al.) |
| Indications for anti-seizure prophylaxis, duration of anti-seizure prophylaxis, agents used for seizure treatment, initiation of anti-epileptic treatment | - |
| Items of the questionnaire (can be found in additional file 1.) with corresponding recommendations in the Brain Trauma Foundation guidelines for the Management of Severe Traumatic Brain Injury (4^th^ edition), (Carney N, Totten AM, O'Reilly C, Ullman JS, Hawryluk GW, Bell MJ, Bratton SL, Chesnut R, Harris OA, Kissoon N et al: Guidelines for the Management of Severe Traumatic Brain Injury, Fourth Edition. Neurosurgery 2017, 80(1):6-15.)  CPP: cerebral perfusion pressure, ICU: intensive care unit, ICP: intracranial pressure, PaCO_2_: partial pressure of carbon dioxide in arterial blood, PaO_2_: partial pressure of oxygen in arterial blood | |
