# Supplementary material for: Variation in general supportive and preventive intensive care management of traumatic brain injury: a survey in 66 neurotrauma centers participating in the Collaborative European NeuroTrauma Effectiveness Research in Traumatic Brain Injury (CENTER-TBI) study
Source: Crit Care. 2018 Apr 13;22:90. doi: 10.1186/s13054-018-2000-6 (PMC5898014; doi:10.1186/s13054-018-2000-6)
Supplement: Supplementary file 5 — CENTER-TBI investigators and participants participating in the CENTER-TBI study and their corresponding affiliations. (DOCX 33 kb) [file 13054_2018_2000_MOESM5_ESM.docx]

**Center-TBI investigators and participants (additional file 5)**

Adams Hadie ^1^, Alessandro Masala ^2^, Allanson Judith ^3^, Amrein Krisztina ^4^, Andaluz Norberto ^5^, Andelic Nada ^6^, Andrea Nanni ^2^, Andreassen Lasse ^7^, Anke Audny ^8^, Antoni Anna ^9^, Ardon Hilko ^10^, Audibert Gérard ^11^, Auslands Kaspars ^12^, Azouvi Philippe ^13^, Baciu Camelia ^14^, Bacon Andrew ^15^, Badenes Rafael ^16^, Baglin Trevor ^17^, Bartels Ronald ^18^, Barzó Pál ^19^, Bauerfeind Ursula ^20^, Beer Ronny ^21^, Belda Francisco Javier ^16^, Bellander Bo‑Michael ^22^, Belli Antonio ^23^, Bellier Rémy ^24^, Benali Habib ^25^, Benard Thierry ^24^, Berardino Maurizio ^26^, Beretta Luigi ^27^, Beynon Christopher ^28^, Bilotta Federico ^16^, Binder Harald ^9^, Biqiri Erta ^14^, Blaabjerg Morten ^29^, Borgen Lund Stine ^30^, Bouzat Pierre ^31^, Bragge Peter ^32^, Brazinova Alexandra ^33^, Brehar Felix ^34^, Brorsson Camilla ^35^, Buki Andras ^36^, Bullinger Monika ^37^, Bučková Veronika ^33^, Calappi Emiliana ^38^, Cameron Peter ^39^, Carbayo Lozano Guillermo ^40^, Carise Elsa ^24^, Carpenter K. ^41^, Castaño‑León Ana M. ^42^, Causin Francesco ^43^, Chevallard Giorgio ^14^, Chieregato Arturo ^14^, Citerio Giuseppe ^44, 45^, Cnossen Maryse ^46^, Coburn Mark Coburn ^47^, Coles Jonathan ^48^, Cooper Jamie D. ^49^, Correia Marta ^50^, Covic Amra ^51^, Curry Nicola ^52^, Czeiter Endre ^53^, Czosnyka Marek ^54^, Dahyot‑Fizelier Claire ^24^, Damas François ^55^, Damas Pierre ^56^, Dawes Helen ^57^, De Keyser Véronique ^58^, Della Corte Francesco ^59^, Depreitere Bart ^60^, Ding Shenghao ^61^, Dippel Diederik ^62^, Dizdarevic Kemal ^63^, Dulière Guy‑Loup ^55^, Dzeko Adelaida ^64^, Eapen George ^15^, Engemann Heiko ^51^, Ercole Ari ^65^, Esser Patrick ^57^, Ezer Erzsébet ^66^, Fabricius Martin ^67^, Feigin Valery L. ^68^, Feng Junfeng ^61^, Foks Kelly ^62^, Fossi Francesca ^14^, Francony Gilles ^31^, Frantzén Janek ^69^, Freo Ulderico ^70^, Frisvold Shirin ^71^, Furmanov Alex ^72^, Gagliardo Pablo ^73^, Galanaud Damien ^25^, Gao Guoyi ^74^, Geleijns Karin ^41^, Ghuysen Alexandre ^75^, Giraud Benoit ^24^, Glocker Ben ^76^, Gomez Pedro A. ^42^, Grossi Francesca ^59^, Gruen Russell L. ^77^, Gupta Deepak ^78^, Haagsma Juanita A. ^46^, Hadzic Ermin ^64^, Haitsma Iain ^79^, Hartings Jed A. ^80^, Helbok Raimund ^21^, Helseth Eirik ^81^, Hertle Daniel ^28^, Hill Sean ^82^, Hoedemaekers Astrid ^83^, Hoefer Stefan ^51^, Hutchinson Peter J. ^1^, Håberg Asta Kristine ^84^, Jacobs Bram ^85^, Janciak Ivan ^86^, Janssens Koen ^58^, Jiang Ji‑yao ^74^, Jones Kelly ^87^, Kalala Jean‑Pierre ^88^, Kamnitsas Konstantinos ^76^, Karan Mladen ^89^, Karau Jana ^20^, Katila Ari ^69^, Kaukonen Maija ^90^, Keeling David ^52^, Kerforne Thomas ^24^, Ketharanathan Naomi ^41^, Kettunen Johannes ^91^, Kivisaari Riku ^90^, Kolias Angelos G. ^1^, Kolumbán Bálint ^92^, Kompanje Erwin ^93^, Kondziella Daniel ^67^, Koskinen Lars‑Owe ^35^, Kovács Noémi ^92^, Kálovits Ferenc ^94^, Lagares Alfonso ^42^, Lanyon Linda ^82^, Laureys Steven ^95^, Lauritzen Martin ^67^, Lecky Fiona ^96^, Ledig Christian ^76^, Lefering Rolf ^97^, Legrand Valerie ^98^, Lei Jin ^61^, Levi Leon ^99^, Lightfoot Roger ^100^, Lingsma Hester ^46^, Loeckx Dirk ^101^, Lozano Angels ^16^, Luddington Roger ^17^, Luijten‑Arts Chantal ^83^, Maas Andrew I.R. ^58^, MacDonald Stephen ^17^, MacFayden Charles ^65^, Maegele Marc ^102^, Majdan Marek ^33^, Major Sebastian ^103^, Manara Alex ^104^, Manhes Pauline ^31^, Manley Geoffrey ^105^, Martin Didier ^106^, Martino Costanza ^2^, Maruenda Armando ^16^, Maréchal Hugues ^55^, Mastelova Dagmara ^86^, Mattern Julia ^28^, McMahon Catherine ^107^, Melegh Béla ^108^, Menon David ^65^, Menovsky Tomas ^58^, Morganti‑Kossmann Cristina ^109^, Mulazzi Davide ^38^, Mutschler Manuel ^102^, Mühlan Holger ^110^, Negru Ancuta ^111^, Nelson David ^82^, Neugebauer Eddy ^102^, Newcombe Virginia ^65^, Noirhomme Quentin ^95^, Nyirádi József ^4^, Oddo Mauro ^112^, Oldenbeuving Annemarie ^113^, Oresic Matej ^114^, Ortolano Fabrizio ^38^, Palotie Aarno ^91, 115, 116^, Parizel Paul M. ^117^, Patruno Adriana ^118^, Payen Jean‑François ^31^, Perera Natascha ^119^, Perlbarg Vincent ^25^, Persona Paolo ^120^, Peul Wilco ^121^, Pichon Nicolas ^122^, Piilgaard Henning ^67^, Piippo Anna ^90^, Pili Floury Sébastien ^123^, Pirinen Matti ^91^, Ples Horia ^111^, Polinder Suzanne ^46^, Pomposo Inigo ^40^, Psota Marek ^33^, Pullens Pim ^117^, Puybasset Louis ^124^, Ragauskas Arminas ^125^, Raj Rahul ^90^, Rambadagalla Malinka ^126^, Rehorčíková Veronika ^33^, Rhodes Jonathan ^127^, Richardson Sylvia ^128^, Ripatti Samuli ^91^, Rocka Saulius ^125^, Rodier Nicolas ^122^, Roe Cecilie ^129^, Roise Olav ^130^, Roks Gerwin ^131^, Romegoux Pauline ^31^, Rosand Jonathan ^132^, Rosenfeld Jeffrey ^109^, Rosenlund Christina ^133^, Rosenthal Guy ^72^, Rossaint Rolf ^47^, Rossi Sandra ^120^, Rostalski Tim ^110^, Rueckert Daniel ^76^, Ruiz de Arcaute Felix ^101^, Rusnák Martin ^86^, Sacchi Marco ^14^, Sahakian Barbara ^65^, Sahuquillo Juan ^134^, Sakowitz Oliver ^135, 136^, Sala Francesca ^118^, Sanchez‑Pena Paola ^25^, Sanchez‑Porras Renan ^28, 135^, Sandor Janos ^137^, Santos Edgar ^28^, Sasse Nadine ^51^, Sasu Luminita ^59^, Savo Davide ^118^, Schipper Inger ^138^, Schlößer Barbara ^20^, Schmidt Silke ^110^, Schneider Annette ^97^, Schoechl Herbert ^139^, Schoonman Guus ^131^, Schou Rico Frederik ^140^, Schwendenwein Elisabeth ^9^, Schöll Michael ^28^, Sir Özcan ^141^, Skandsen Toril ^142^, Smakman Lidwien ^143^, Smeets Dirk ^101^, Smielewski Peter ^54^, Sorinola Abayomi ^144^, Stamatakis Emmanuel ^65^, Stanworth Simon ^52^, Stegemann Katrin ^110^, Steinbüchel Nicole ^145^, Stevens Robert ^146^, Stewart William ^147^, Steyerberg Ewout W. ^46^, Stocchetti Nino ^148^, Sundström Nina ^35^, Synnot Anneliese ^149, 150^, Szabó József ^94^, Söderberg Jeannette ^82^, Taccone Fabio Silvio ^16^, Tamás Viktória ^144^, Tanskanen Päivi ^90^, Tascu Alexandru ^34^, Taylor Mark Steven ^33^, Te  Ao Braden ^68^, Tenovuo Olli ^69^, Teodorani Guido ^151^, Theadom Alice ^68^, Thomas Matt ^104^, Tibboel Dick ^41^, Tolias Christos ^152^, Tshibanda Jean‑Flory Luaba ^153^, Tudora Cristina Maria ^111^, Vajkoczy Peter ^154^, Valeinis Egils ^155^, Van Hecke Wim ^101^, Van Praag Dominique ^58^, Van Roost Dirk ^88^, Van Vlierberghe Eline ^101^, Vande Vyvere Thijs ^101^, Vanhaudenhuyse Audrey ^25, 95^, Vargiolu Alessia ^118^, Vega Emmanuel ^156^, Verheyden Jan ^101^, Vespa Paul M. ^157^, Vik Anne ^158^, Vilcinis Rimantas ^159^, Vizzino Giacinta ^14^, Vleggeert‑Lankamp Carmen ^143^, Volovici Victor ^79^, Vulekovic Peter ^89^, Vámos Zoltán ^66^, Wade Derick ^57^, Wang Kevin K.W. ^160^, Wang Lei ^61^, Wildschut Eno ^41^, Williams Guy ^65^, Willumsen Lisette ^67^, Wilson Adam ^5^, Wilson Lindsay ^161^, Winkler Maren K.L. ^103^, Ylén Peter ^162^, Younsi Alexander ^28^, Zaaroor Menashe ^99^, Zhang Zhiqun ^163^, Zheng Zelong ^28^, Zumbo Fabrizio ^2^, de Lange Stefanie ^97^, de Ruiter Godard C.W. ^143^, den Boogert Hugo ^18^, van Dijck Jeroen ^164^, van Essen Thomas A. ^121^, van Heugten Caroline ^57^, van der Jagt Mathieu ^165^, van der Naalt Joukje ^85^

^1^ Division of Neurosurgery, Department of Clinical Neurosciences, Addenbrooke’s Hospital & University of Cambridge, Cambridge, UK

^2^ Department of Anesthesia & Intensive Care,M. Bufalini Hospital, Cesena, Italy

^3^ Department of Clinical Neurosciences, Addenbrooke’s Hospital & University of Cambridge, Cambridge, UK

^4^ János Szentágothai Research Centre, University of Pécs, Pécs, Hungary

^5^ University of Cincinnati, Cincinnati, Ohio, United States

^6^ Division of Surgery and Clinical Neuroscience, Department of Physical Medicine and Rehabilitation, Oslo University Hospital and University of Oslo, Oslo, Norway

^7^ Department of Neurosurgery, University Hospital Northern Norway, Tromso, Norway

^8^ Department of Physical Medicine and Rehabilitation, University hospital Northern Norway

^9^ Trauma Surgery, Medical University Vienna, Vienna, Austria

^10^ Department of Neurosurgery, Elisabeth-Tweesteden Ziekenhuis, Tilburg, the Netherlands

^11^ Department of Anesthesiology & Intensive Care, University Hospital Nancy, Nancy, France

^12^ Riga Eastern Clinical University Hospital, Riga, Latvia

^13^ Raymond Poincare hospital, Assistance Publique – Hopitaux de Paris, Paris, France

^14^ NeuroIntensive Care, Niguarda Hospital

^15^ Neurointensive Care , Sheffield Teaching Hospitals NHS Foundation Trust, Sheffield, UK

^16^ Department Anesthesiology and Surgical-Trauma Intensive Care, Hospital Clinic Universitari de Valencia, Spain

^17^ Cambridge University Hospitals, Cambridge, UK

^18^ Department of Neurosurgery, Radboud University Medical Center

^19^ Department of Neurosurgery, University of Szeged, Szeged, Hungary

^20^ Institute for Transfusion Medicine (ITM) , Witten/Herdecke University, Cologne, Germany

^21^ Department of Neurocritical care, Innsbruck Medical University, Innsbruck, Austria

^22^ Deparment of Neurosurgery & Anesthesia & intensive care medicine, Karolinska University Hospital, Stockholm, Sweden

^23^ NIHR Surgical Reconstruction and Microbiology Research Centre, Birmingham, UK

^24^ Intensive care Unit, CHU Poitiers, Poitiers, France

^25^ Anesthesie-Réanimation, Assistance Publique – Hopitaux de Paris, Paris, France

^26^ Department of Anesthesia & ICU, AOU Città della Salute e della Scienza di Torino - Orthopedic and Trauma Center, Torino, Italy

^27^ Department of Anesthesiology & Intensive Care, S Raffaele University Hospital, Milan, Italy

^28^ Department of Neurosurgery, University Hospital Heidelberg, Heidelberg, Germany

^29^ Department of Neurology, Odense University Hospital, Odense, denmark

^30^ Departments of Neuroscience and  Nursing Science, Norwegian University of Science and Technology, Trondheim, Norway

^31^ Department of Anesthesiology & Intensive Care, University Hospital of Grenoble, Grenoble, France

^32^ BehaviourWorks Australia, Monash Sustainability Institute, Monash University, Victoria, Australia

^33^ Department of Public Health, Faculty of Health Sciences and Social Work, Trnava University, Trnava, Slovakia

^34^ Department of Neurosurgery, Bagdasar-Arseni Emergency Clinical Hospital, Bucharest, Romania

^35^ Department of Neurosurgery, Umea University Hospital, Umea, Sweden

^36^ Department of Neurosurgery, University of Pecs and MTA-PTE Clinical Neuroscience MR Research Group and Janos Szentagothai Research Centre, University of Pecs, Hungarian Brain Research Program, Pecs, Hungary

^37^ Department of Medical Psychology, Universitätsklinikum Hamburg-Eppendorf, Hamburg, Germany

^38^ Neuro ICU, Fondazione IRCCS Cà Granda Ospedale Maggiore Policlinico, Milan, Italy

^39^ Department of Epidemiology and Preventive Medicine, Monash University, Melbourne, Victoria, Australia

^40^ Department of Neurosurgery, Hospital of Cruces, Bilbao, Spain

^41^ Intensive Care and Department of Pediatric Surgery, Erasmus Medical Center, Sophia Children’s Hospital, Rotterdam, The Netherlands

^42^ Department of Neurosurgery, Hospital Universitario 12 de Octubre, Madrid, Spain

^43^ Department of Neuroscience, Azienda Ospedaliera Università di Padova, Padova, Italy

^44^ NeuroIntensive Care, Azienda Ospedaliera San Gerardo di Monza, Monza, Italy

^45^ School of Medicine and Surgery, Università Milano Bicocca, Milano, Italy

^46^ Department of Public Health, Erasmus Medical Center-University Medical Center, Rotterdam, The Netherlands

^47^ Department of Anaesthesiology, University Hospital of Aachen, Aachen, Germany

^48^ Department of Anesthesia & Neurointensive Care, Cambridge Universiyt Hospital NHS Foundation Trust, Cambridge, UK

^49^ School of Public Health & PM, Monash University and The Alfred Hospital, Melbourne, Victoria, Australia

^50^ Radiology/MRI department, MRC Cognition and Brain Sciences Unit, Cambridge, UK

^51^ Institute of Medical Psycholology and Medical Sociology, Universitätsmedizin Göttingen, Göttingen, Germany

^52^ Oxford University Hospitals NHS Trust, Oxford, UK

^53^ Department of Neurosurgery, University of Pecs and MTA-PTE Clinical Neuroscience MR Research Group and Janos Szentagothai Research Centre, University of Pecs, Hungarian Brain Research Program (Grant No. KTIA 13 NAP-A-II/8), Pecs, Hungary

^54^ Brain Physics Lab, Division of Neurosurgery, Dept of Clinical Neurosciences, University of Cambridge, Addenbrooke’s Hospital, Cambridge, UK

^55^ Intensive Care Unit, CHR Citadelle , Liège, Belgium

^56^ Intensive Care Unit, CHU , Liège, Belgium

^57^ Movement Science Group, Faculty of Health and Life Sciences, Oxford Brookes University, Oxford, UK

^58^ Department of Neurosurgery, Antwerp University Hospital and University of Antwerp, Edegem, Belgium

^59^ Department of Anesthesia & Intensive Care, Maggiore Della Carità Hospital, Novara, Italy

^60^ Department of Neurosurgery, University Hospitals Leuven, Leuven, Belgium

^61^ Department of Neurosurgery, Renji Hospital, Shanghai Jiaotong University School of Medicine, Shanghai, China

^62^ Department of Neurology, Erasmus MC, Rotterdam, the Netherlands

^63^ Department of Neurosurgery, Medical Faculty and clinical center University of Sarajevo, Sarajevo, Bosnia Herzegovina

^64^ Department of Neurosurgery, Regional Medical Center dr Safet Mujić, Mostar, Bosnia Herzegovina

^65^ Division of Anaesthesia, University of Cambridge, Addenbrooke’s Hospital, Cambridge, UK

^66^ Department of Anaesthesiology and Intensive Therapy, University of Pécs, Pécs, Hungary

^67^ Departments of Neurology, Clinical Neurophysiology and Neuroanesthesiology, Region Hovedstaden Rigshospitalet, Copenhagen, Denmark

^68^ National Institute for Stroke and Applied Neurosciences, Faculty of Health and Environmental Studies, Auckland University of Technology, Auckland, New Zealand

^69^ Rehabilitation and Brain Trauma, Turku University Central Hospital and University of Turku, Turku, Finland

^70^ Department of Medicine, Azienda Ospedaliera Università di Padova, Padova, Italy

^71^ Department of Anesthesiology and Intensive care, University Hospital Northern Norway, Tromso, Norway

^72^ Department of Neurosurgery, Hadassah-hebrew University Medical center, Jerusalem, Israel

^73^ Fundación Instituto Valenciano de Neurorrehabilitación (FIVAN), Valencia, Spain

^74^ Department of Neurosurgery, Shanghai Renji hospital, Shanghai Jiaotong University/school of medicine, Shanghai, China

^75^ Emergency Department, CHU , Liège, Belgium

^76^ Department of Computing, Imperial College London, London, UK

^77^ Lee Kong Chian School of Medicine, Nanyang Technological University, Singapore; and Monash University, Australia

^78^ Department of Neurosurgery, Neurosciences Centre & JPN Apex trauma centre, All India Institute of Medical Sciences, New Delhi-110029, India

^79^ Department of Neurosurgery, Erasmus MC, Rotterdam, the Netherlands

^80^ Department of Neurosurgery, University of Cincinnati, Cincinnati, Ohio, USA

^81^ Department of Neurosurgery, Oslo University Hospital, Oslo, Norway

^82^  Department of Physiology and Pharmacology, Section of Perioperative Medicine and Intensive Care, Karolinska Institutet, Stockholm, Sweden

^83^ Department of Intensive Care Medicine, Radboud University Medical Center

^84^ Department of Medical Imaging, St. Olavs Hospital and Department of Neuroscience, Norwegian University of Science and Technology, Trondheim, Norway

^85^ Department of Neurology, University Medical Center Groningen, Groningen, Netherlands

^86^ International Neurotrauma Research Organisation, Vienna, Austria

^87^ National Institute for Stroke & Applied Neurosciences of the AUT University, Auckland, New Zealand

^88^ Department of Neurosurgery, UZ Gent, Gent, Belgium

^89^ Department of Neurosurgery, Clinical centre of Vojvodina, Novi Sad, Serbia

^90^ Helsinki University Central Hospital

^91^ Institute for Molecular Medicine Finland, University of Helsinki, Helsinki, Finland

^92^ Hungarian Brain Research Program - Grant No. KTIA 13 NAP-A-II/8, University of Pécs, Pécs, Hungary

^93^ Department of Intensive Care and Department of Ethics and Philosophy of Medicine, Erasmus Medical Center, Rotterdam, The Netherlands

^94^ Department of Neurological & Spinal Surgery, Markusovszky University Teaching Hospital, Szombathely, Hungary

^95^ Cyclotron Research Center , University of Liège, Liège, Belgium

^96^ Emergency Medicine Research in Sheffield, Health Services Research Section, School of Health and Related Research (ScHARR), University of Sheffield, Sheffield ,UK

^97^ Institute of Research in Operative Medicine (IFOM) , Witten/Herdecke University, Cologne, Germany

^98^ VP Global Project Management CNS, ICON, Paris, France

^99^ Department of Neurosurgery, Rambam Medical Center, Haifa, Israel

^100^ Department of Anesthesiology & Intensive Care, University Hospitals Southhampton NHS Trust, Southhampton, UK

^101^ icoMetrix NV, Leuven, Belgium

^102^ Cologne-Merheim Medical Center (CMMC), Department of Traumatology, Orthopedic Surgery and Sportmedicine, Witten/Herdecke University, Cologne, Germany

^103^ Centrum für Schlaganfallforschung, Charité – Universitätsmedizin Berlin, Berlin, Germany

^104^ Intensive Care Unit, Southmead Hospital, Bristol, Bristol, UK

^105^ Department of Neurological Surgery, University of California, San Francisco, California, USA

^106^ Department of Neurosurgery, CHU , Liège, Belgium

^107^ Department of Neurosurgery, The Walton centre NHS Foundation Trust, Liverpool, UK

^108^ Department of Medical Genetics, University of Pécs, Pécs, Hungary

^109^ National Trauma Research Institute, The Alfred Hospital, Monash University, Melbourne, Victoria, Australia

^110^ Department Health and Prevention, University Greifswald, Greifswald, Germany

^111^ Department of Neurosurgery, Emergency County Hospital Timisoara , Timisoara, Romania

^112^ Centre Hospitalier Universitaire Vaudois

^113^ Department of Intensive Care, Elisabeth-Tweesteden Ziekenhuis, Tilburg, the Netherlands

^114^ Department of Systems Medicine, Steno Diabetes Center, Gentofte, Denmark

^115^ Analytic and Translational Genetics Unit, Department of Medicine; Psychiatric & Neurodevelopmental Genetics Unit, Department of Psychiatry; Department of Neurology, Massachusetts General Hospital, Boston, MA, USA

^116^ Program in Medical and Population Genetics; The Stanley Center for Psychiatric Research, The Broad Institute of MIT and Harvard, Cambridge, MA, USA

^117^ Department of Radiology, Antwerp University Hospital and University of Antwerp, Edegem, Belgium

^118^ NeuroIntenisve Care Unit, Department of Anesthesia & Intensive Care Azienda Ospedaliera San Gerardo di Monza, Monza, Italy

^119^ International Projects Management, ARTTIC, Munchen, Germany

^120^ Department of Anesthesia & Intensive Care, Azienda Ospedaliera Università di Padova, Padova, Italy

^121^ Dept. of Neurosurgery, Leiden University Medical Center, Leiden, The Netherlands and Dept. of Neurosurgery, Medical Center Haaglanden, The Hague, The Netherlands

^122^ Intensive Care Unit, CHU Dupuytren, Limoges, France

^123^ Intensive Care Unit, CHRU de Besançon, Besançon, France

^124^ Department of Anesthesiology and Critical Care, Pitié -Salpêtrière Teaching Hospital, Assistance Publique, Hôpitaux de Paris and University Pierre et Marie Curie, Paris, France

^125^ Department of Neurosurgery, Kaunas University of technology and Vilnius University, Vilnius, Lithuania

^126^ Rezekne Hospital, Latvia

^127^ Department of Anaesthesia, Critical Care & Pain MedicineNHS Lothian & University of Edinburg, Edinburgh, UK

^128^ Director, MRC Biostatistics Unit, Cambridge Institute of Public Health, Cambridge, UK

^129^ Department of Physical Medicine and Rehabilitation, Oslo University Hospital/University of Oslo, Oslo, Norway

^130^ Division of Surgery and Clinical Neuroscience, Oslo University Hospital, Oslo, Norway

^131^ Department of Neurology, Elisabeth-TweeSteden Ziekenhuis, Tilburg, the Netherlands

^132^ Broad Institute, Cambridge MA Harvard Medical School, Boston MA, Massachusetts General Hospital, Boston MA, USA

^133^ Department of Neurosurgery, Odense University Hospital, Odense, Denmark

^134^ Department of Neurosurgery, Vall d'Hebron University Hospital, Barcelona, Spain

^135^ Klinik für Neurochirurgie, Klinikum Ludwigsburg, Ludwigsburg, Germany

^136^ University Hospital Heidelberg, Heidelberg, Germany

^137^ Division of Biostatistics and Epidemiology, Department of Preventive Medicine, University of Debrecen, Debrecen, Hungary

^138^ Department of Traumasurgery, Leiden University Medical Center, Leiden, The Netherlands

^139^ Department of Anaesthesiology and Intensive Care, AUVA Trauma Hospital, Salzburg, Austria

^140^ Department of Neuroanesthesia and Neurointensive Care, Odense University Hospital, Odense, Denmark

^141^ Department of Emergency Care Medicine, Radboud University Medical Center

^142^ Department of Physical Medicine and Rehabilitation, St.Olavs Hospital and and Department of Neuroscience, Norwegian University of Science and Technology, Trondheim, Norway

^143^ Neurosurgical Cooperative Holland, Department of Neurosurgery, Leiden University Medical Center and Medical Center Haaglanden, Leiden and The Hague, The Netherlands

^144^ Department of Neurosurgery, University of Pécs, Pécs, Hungary

^145^ Universitätsmedizin Göttingen, Göttingen, Germany

^146^ Division of Neuroscience Critical Care, John Hopkins University School of Medicine, Baltimore, USA

^147^ Department of Neuropathology, Queen Elizabeth University Hospital and University of Glasgow , Glasgow, UK

^148^ Department of Pathophysiology and Transplantation, Milan University, and Neuroscience ICU, Fondazione IRCCS Cà Granda Ospedale Maggiore Policlinico, Milano, Italy

^149^ Australian & New Zealand Intensive Care Research Centre, Department of Epidemiology and Preventive Medicine, School of Public Health and Preventive Medicine, Monash University, Melbourne, Australia

^150^ Cochrane Consumers and Communication Review Group, Centre for Health Communication and Participation, School of Psychology and Public Health, La Trobe University, Melbourne, Australia

^151^ Department of Reahabilitation, M. Bufalini Hospital, Cesena, Italy

^152^ Department of Neurosurgery, Kings college London, London, UK

^153^ Radiology/MRI Department, CHU , Liège, Belgium

^154^ Neurologie, Neurochirurgie und Psychiatrie, Charité – Universitätsmedizin Berlin, Berlin, Germany

^155^ Pauls Stradins Clinical University Hospital, Riga, Latvia

^156^ Department of Anesthesiology-Intensive Care, Lille University Hospital, Lille, France

^157^ Director of Neurocritical Care, University of California, Los Angeles, USA

^158^ Department of Neurosurgery, St.Olavs Hospital and Department of Neuroscience, Norwegian University of Science and Technology, Trondheim, Norway

^159^ Department of Neurosurgery, Kaunas University of Health Sciences, Kaunas, Lithuania

^160^ Department of Psychiatry, University of Florida, Gainesville, Florida, USA

^161^ Division of Psychology, University of Stirling, Stirling, UK

^162^ VTT Technical Research Centre, Tampere, Finland

^163^ University of Florida, Gainesville, Florida, USA

^164^ Department of Neurosurgery, The HAGA Hospital, The Hague, The Netherlands

^165^ Department of Intensive Care, Erasmus MC, Rotterdam, the Netherlands
